# The cacao procyanidin extract-caused anti-hyperglycemic effect was changed by the administration timings

**DOI:** 10.3164/jcbn.20-45

**Published:** 2020-06-05

**Authors:** Ken-yu Hironao, Hitoshi Ashida, Yoko Yamashita

**Affiliations:** 1 Department of Agrobioscience, Graduate School of Agricultural Science, Kobe University, 1-1 Rokkodai-cho, Nada-ku, Kobe 657-8501, Japan

**Keywords:** cacao liquor procyanidin rich extract, circadian rhythm, hyperglycemia, timing, muscle

## Abstract

Mammals have the biological clocks with approximately 24 h-rhythm. Energy metabolism including glucose metabolism is regulated by the biological clocks. Glucose metabolism is affected by not only meal volume and its energy but also meal timing. We have reported that cacao liquor procyanidin-rich extract (CLPr) ameliorated the postprandial hyperglycemia through AMP-activated protein kinase pathway. However, the effect of administration timing of CLPr on the postprandial hyperglycemia and its signaling pathway are still unclear. In the present study, we compared the effect of CLPr-administration at the rest-phase (light-period) and active-phase (dark-period) on glucose metabolism. Single oral administration of CLPr to ICR mice at the rest-phase, but not at the active-phase, promoted phosphorylation of AMP-activated protein kinase and its upstream liver kinase B1 and translocation of glucose transporter 4 to the plasma membrane in the skeletal muscle, resulting in reduced postprandial hyperglycemia. These results indicated that the intake of CLPr at the rest-phase more effectively suppressed postprandial hyperglycemia.

## Introduction

Mammals have the biological clocks with approximately 24 h-rhythm and the synchronization of rhythm is a vital event for maintaining healthy body functions. The timing of many physiological processes including energy metabolism are coordinated by the circadian system.^(1,2)^ Glucose metabolism is affected by not only meal volume and its energy but also meal timing. For example, postprandial glucose level is affected by meal timing: elevated postprandial glucose level is higher in the evening than in the morning in human.^(3,4)^ Moreover, it has been observed the time-of-day variations in glucose tolerance with a peak in the morning and a bottom in the evening/night.^(4–7)^ These alteration are due to the variations of digestion, absorption, and metabolism of glucose in various tissues under regulating by the circadian system.^(1,4)^

Among the peripheral tissues, skeletal muscle is the most important tissue for maintain of a postprandial glucose homeostasis, because approximately 80% of insulin-stimulated glucose uptake is accounted for by muscle.^(8)^ In the skeletal muscle, glucose transporter type 4 (GLUT4), which is specifically expressing in skeletal and cardiac muscle and adipose tissue, plays pivotal role in the glucose uptake after translocation from intracellular storage vesicles to the plasma membrane stimulated by insulin stimulus and muscle contraction.^(9)^ Muscle contraction and exercise increase the cellular AMP/ATP ratio, resulting in promotion of adenosine monophosphate-activated protein kinase (AMPK) phosphorylation.^(10–12)^ AMPK is known as a nutrient and energy sensor that regulates various energy metabolism including glucose metabolism.^(11,13)^ Therefore, AMPK is a target molecule for prevention of postprandial hyperglycemia.

Recently, much attention has been paid to the health beneficial functions of polyphenols and polyphenol-rich food materials.^(14)^ Prevention of hyperglycemia by polyphenols by promoting translocation of GLUT4 through the activation of AMPK signaling pathway in the muscle cells is well documented.^(15,16)^ Of these, procyanidin has a unique mechanism for promotion of GLUT4 translocation, because it activates insulin signaling pathway through secretion of GLP-1, in addition to the activation of AMPK pathway.

Recently, we have previously reported that a cacao liquor procyanidin-rich extract (CLPr) derived from Cacao beans (*Theobroma cacao*) suppressed hyperglycemia accompanied by GLUT4 translocation through the activation of both AMPK and GLP-1-dependent insulin pathways.^(17)^ Moreover, the results from our more recent report demonstrate that an antagonist for GLP-1 receptor Exendin (9-39) cancels CLPr-promoted AMPK phosphorylation.^(18)^ In the same report, we have found that CLPr regulates the circadian clock gene expression through the GLP-1 signaling pathway. It is reported that AMPK down-regulates expression of *Bmal1*, which is one of the core clock genes.^(18)^ These results suggest that anti-hyperglycemic effect of CLPr is associate with circadian rhythm. It is poorly understood that the effects of timing of polyphenol ingestion on the preventing postprandial hyperglycemia. Recently, Takahashi *et al.*^(4)^ reported that an intake of EGCG, a major polyphenol in green tea, in the evening effectively suppressed elevation of postprandial glucose level than in the morning in mice and human trial. However, this report did not address to clarify the underlying mechanism. Therefore, in the present study, we compared the effect of the administration timing of CLPr at the early time in a light and dark period on AMPK phosphorylation and followed by GLUT4 translocation and anti-hyperglycemic effect in ICR mice.

## Materials and Methods

### Reagents

CLPr was prepared from cacao liquor and its composition was previously described.^(17,19)^ Glucose was measured using a commercially available kit [Labassay Glucose Wako kit (FUJIFILM Wako Pure Chemical Co., Ltd., Osaka, Japan)]. Antibodies against β-actin, AMPKα, phospho-AMPKα (thr172), phospho-CaMKK2 (ser511), glyceraldehyde-3-phosphate dehydrogenase (GAPDH), liver kinase B1 (LKB1) and phospho-LKB1 (ser428) were purchased from Cell Signaling Technology Co. (Denver, MA). Antibodies against CaMKK2 and GLUT4 were purchased from Abcam (Hercules, CA). Horseradish peroxidase (HRP)-conjugated anti-mouse IgG, HRP-conjugated anti-goat IgG antibodies, anti-insulin receptor (IR), and anti-Lamin B were from Santa Cruz Biotechnology (Santa Cruz, CA). HRP-conjugated anti-rabbit IgG was from Bio-Rad Laboratories Inc. (Hercules, CA). All other reagents used were of the highest grade available from commercial sources.

### Animal experiment

All animal experiments were approved by the Institutional Animal Care and Use Committee (permission no. 27-05-08) and carried out according to the guidelines for animal experiments at Kobe University. Male ICR mice (6 weeks old) were obtained from Japan SLC Inc. and were maintained in a temperature-controlled room (23 ± 2°C) with a 12 h-12 h light-dark cycle [lights on at 8:00 am: equal to Zeitgeber time (ZT) 0]. The mice were allowed free access to tap water and commercial chow [D10012M (AIN-93M base diet, Research Diets, Inc., New Brunswick, NJ)] and were acclimatized for a week.

To examine the effect of CLPr administration on phosphorylation of AMPK and its relating signaling pathways, thirty mice were divided into six groups of five each. Three groups of mice were orally given CLPr in water at 50 or 150 mg/kg body weight or water alone (5 ml/kg body weight) as a vehicle control at ZT 1, and the remaining three groups were given the same dose of CLPr or water at ZT 13. The mice were sacrificed under anesthesia using sevoflurane as an inhalational anesthetic and sodium pentobarbital as an analgesic, and euthanized by exsanguination from cardiac puncture. Gastrocnemius muscle was collected and stored at –80°C until use.

For the oral glucose tolerance test (OGTT), another thirty mice were divided into six groups of five six each. Dosage and administration timing of CLPr were the same as Experiment 1. Sixty min after the CLPr-administration, glucose at 1.0 g/kg body weight was orally administered to the mice. Blood was collected from the tail vein in a heparinized tube at 0 (before glucose load), 15, 30, 60 and 120 min after the glucose load. Blood was centrifuged at 3,000 × *g* for 10 min at 4°C, and resultant supernatant was used as plasma for the measurement of glucose.

### Western blot analysis

Muscle on the tissue lysate, plasma membrane and nuclear fraction were prepared according to the previous reports.^(20–22)^ After protein concentration in the lysate was quantified by a Lowry’s method,^(23)^ the lysate was subjected to western blot analysis following the sodium dodecyl sulfate polyacrylamide gel electrophoresis using a 10% gel. The separated proteins in the gel were transferred onto a polyvinylidene fluoride membrane. The membrane was incubated with the commercially available blocking solutions [Blocking One (for detection of unphosphorylated proteins) and Blocking One-P (for detection of phosphoproteins)] and treated with primary antibodies overnight at 4°C, followed by the corresponding horseradish peroxidase-conjugated secondary antibody for 1 h at room temperature. Protein bands were visualized using Immuno Star LD Western Blotting Substrate and detected with Light-Capture II (ATTO, Tokyo, Japan). The density of the specific band was determined using ImageJ image analysis software (National Institutes of Health, Bethesda, MD).

### Statistical analysis

Statistical analysis was performed with JMP statistical software ver. 11.2.0 (SAS Institute, Cary, NC). Data are represented as the means and SE. The statistical significance of experimental observations was determined using Dunnett’s multiple comparison test, Tukey Kramer multiple comparison test and Student’s *t* test. The level of significance was set as *p*<0.05.

## Results

### Effect of CLPr-administration at different timings on AMPK phosphorylation and its upstream event in the skeletal muscle of mice

**Fig. 1:**
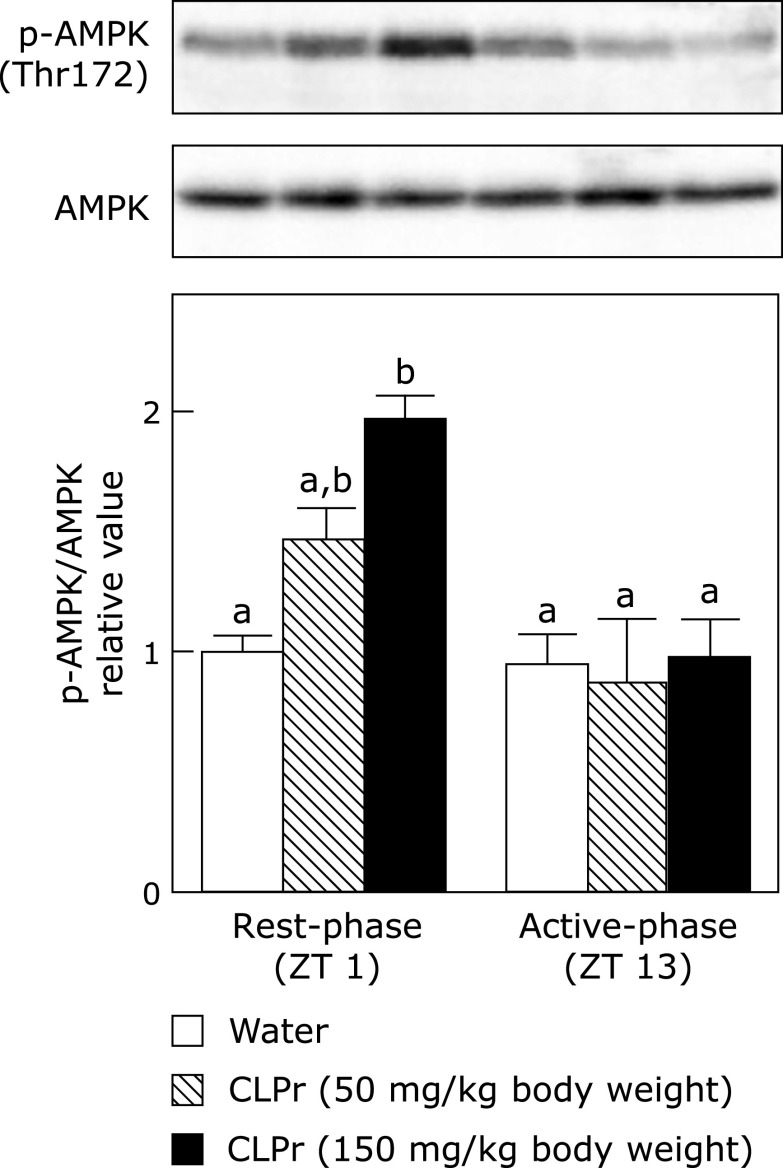
The effect of CLPr administration at ZT 1 or ZT 13 on AMPK phosphorylation in the skeletal muscle of mice. ICR mice were orally administered CLPr at 50 and 150 mg/kg body weight or water (5.0 ml/kg body weight) at ZT1 or 13. The muscle was collected 1 h after the CLPr administration and phosphorylation of AMPK was measured by western blotting. Typical result is shown in the upper panel, while the density of phosphorylation protein after normalized by that of expression protein is shown in the bottom one. Data are represented as the means ± SE (*n* = 5). Different letters indicate significant differences (*p*<0.05 by Tukey-Kramer test).

**Fig. 2:**
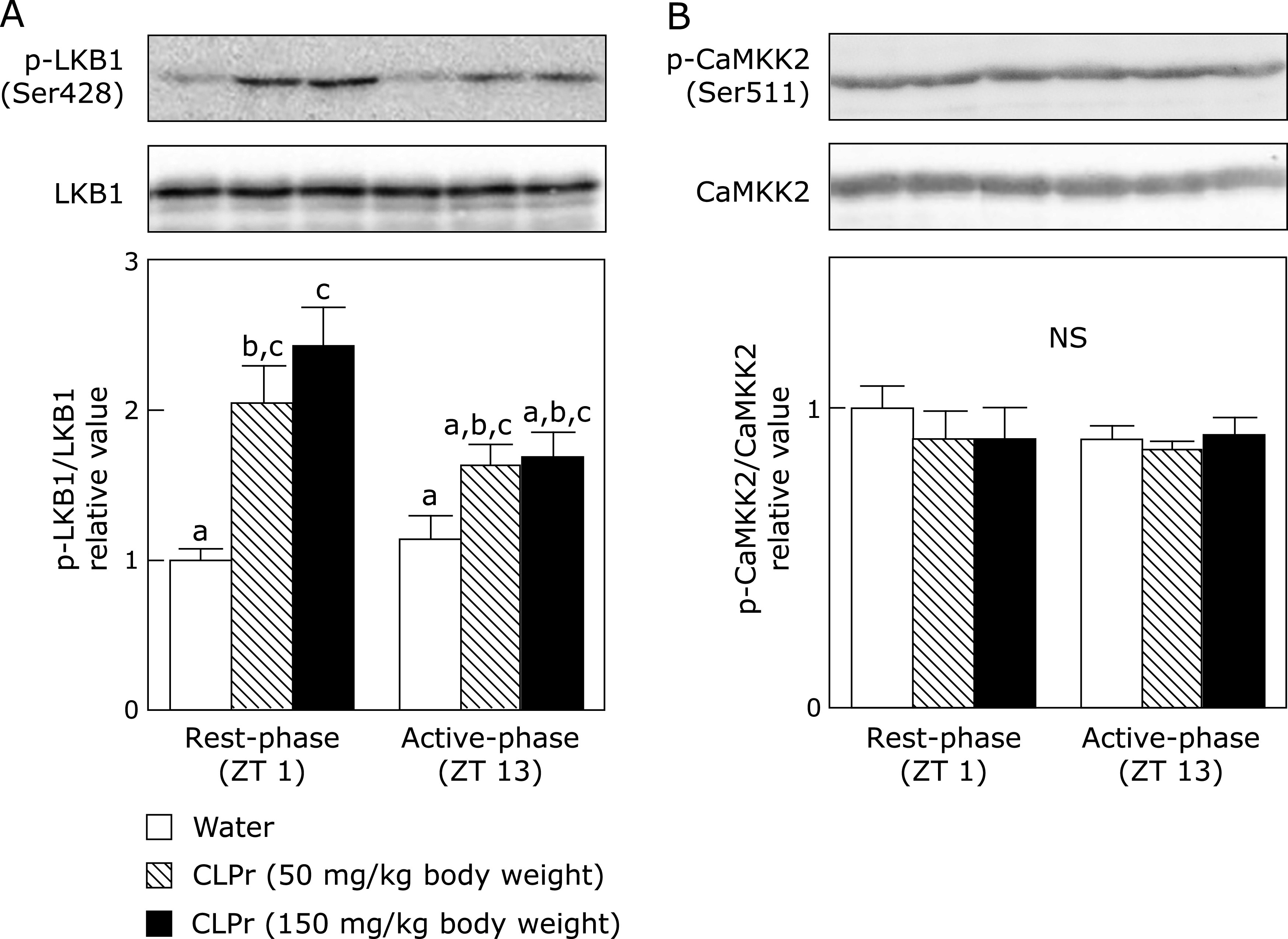
The effect of CLPr administration at ZT 1 or ZT 13 on the LKB1 and CaMKK2 phosphorylation in the skeletal muscle of mice. Animal treatment was the same as in Fig. 1. Phosphorylation of (A) LKB1 and (B) CaMKK2 was measured in the skeletal muscle by western blotting. Typical results are shown in the upper panel, while the density of phosphorylation protein after normalized by that of corresponding expression protein is shown in the bottom one. Data are represented as the means ± SE (*n* = 5). Different letters indicate significant differences (*p*<0.05 by Tukey-Kramer test).

First, we evaluated phosphorylation of AMPK after the administration of CLPr in the rest- and active-phase. As a result, CLPr-administration in the rest-phase, but not in the active-phase, dose-dependently increased AMPK phosphorylation in the skeletal muscle (Fig. 1). It is known that LKB1 and CaMKK2 are mainly acted as the upstream kinases for AMPK phosphorylation.^(24–27)^ It was, therefore, investigated phosphorylation of these kinases after administration of CLPr at different timings. As shown in Fig. 2, CLPr-administration in the rest-phase dose-dependently increased phosphorylation of LKB1 but not that of CaMKK2 in the skeletal muscle. On the other hand, CLPr did not affect phosphorylation of LKB1 in the active-phase (Fig. 2). These results suggested that only CLPr-administration in the rest-phase, but not in the active-phase, mainly activated LKB1/AMPK signaling pathway.

### CLPr affects nucleocytoplasmic transport of LKB1 at ZT 1 in the skeletal muscle of mice

**Fig. 3:**
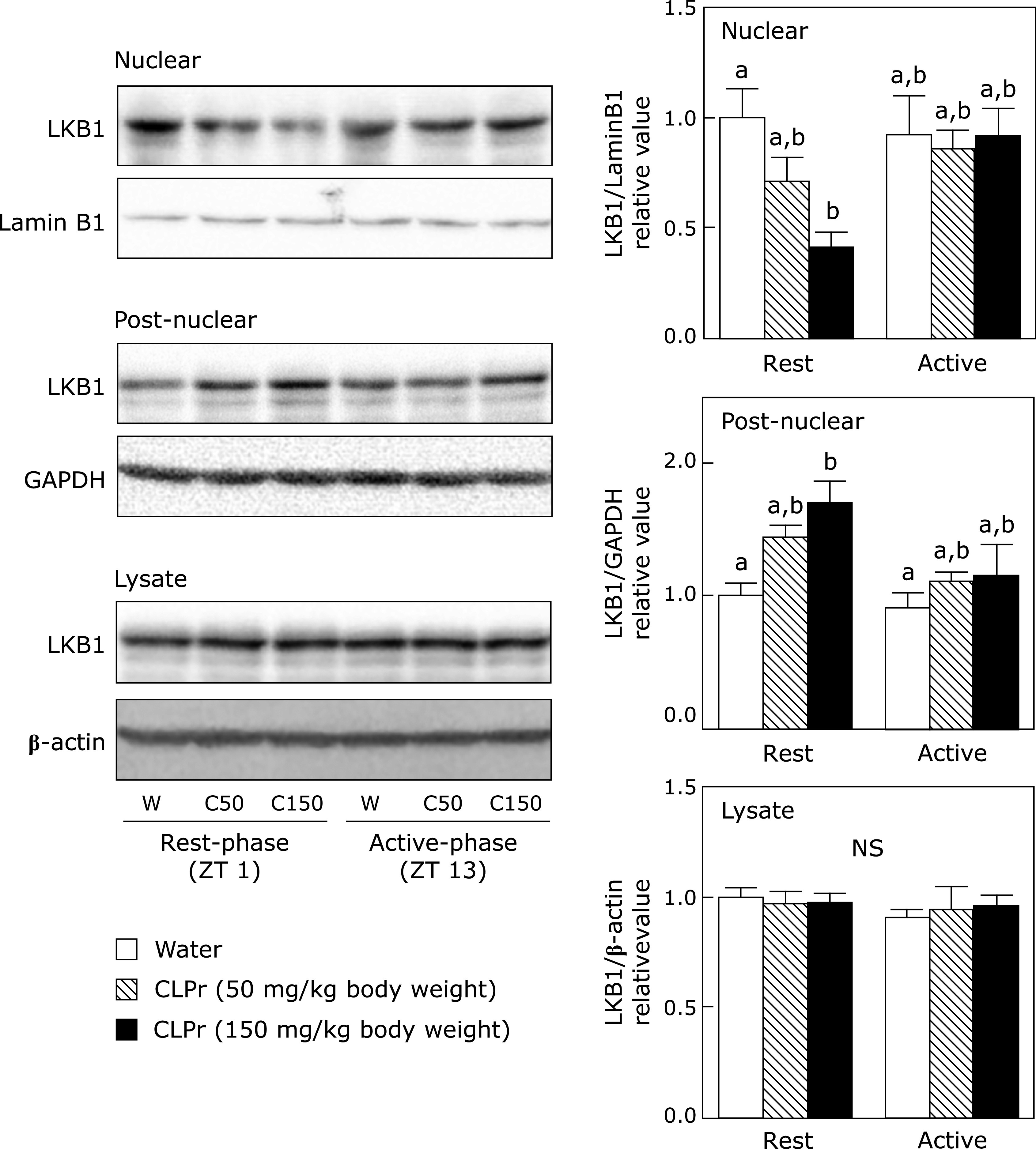
The effect of CLPr administration at ZT 1 or ZT 13 on the LKB1 nucleocytoplasmic transport in the skeletal muscle of mice. Animal treatment was the same as in Fig. 1. The nuclear and post-nuclear fractions, and tissue lysate were prepared from the skeletal muscle and subjected to western blotting analysis for detection of the protein level of the LKB1. Typical results are shown in the upper panel, while the density of LKB1 protein after normalized by that of Lamin B1 (for the nuclear fraction), GAPDH (for the post-nuclear fraction) and β-actin (for tissue lysate) is shown in the bottom one. The results are presented as the mean ± SE (*n* = 5). Different letters indicate significant differences (*p*<0.05 by Tukey-Kramer test).

Since a key step of LKB1 activation is its export from nucleus to the cytoplasm,^(28)^ we investigated the localization of LKB1 in the skeletal muscle after the administration of CLPr. Oral administration of CLPr in the rest-phase dose-dependently decreased the amounts of LKB1 in nucleus, and increased the amounts in the cytoplasm (Fig. 3). In the active-phase, changes in the localization were not observed. Total expression level of LKB1 in the tissue remained unchanged in both administration timings. These results indicated that the key factor of CLPr-caused AMPK activation and its upstream LKB1 phosphorylation and its nucleocytoplasmic transport are influenced by the administration timings.

### CLPr affects GLUT4 translocation to the plasma membrane at ZT 1 in the skeletal muscle

**Fig. 4:**
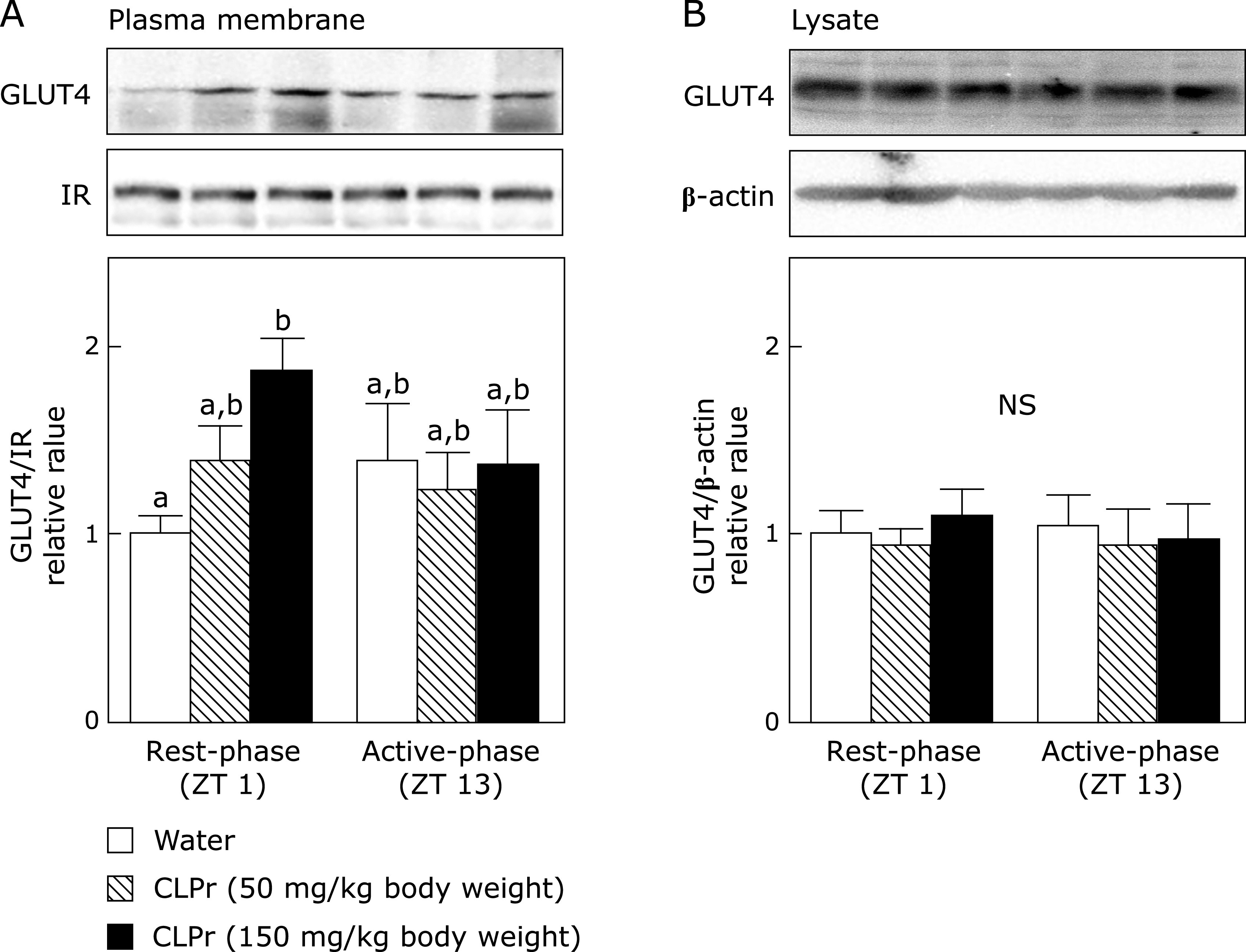
The effect of CLPr administration at ZT 1 or ZT 13 on GLUT4 translocation to the plasma membrane in the skeletal muscle of mice. Animal treatment was the same as in Fig. 1. (A) The plasma membrane fraction and (B) tissue lysate were prepared and subjected to western blotting for estimation of GLUT4 translocation. Typical results are shown in the upper panel, while the density of GLUT4 protein after normalized by that of IR (for the plasma membrane fraction), and β-actin (for tissue lysate) is shown in the bottom one. The results are presented as the mean ± SE (*n* = 5). Different letters indicate significant differences (*p*<0.05 by Tukey-Kramer test).

It is known that AMPK signaling pathway is one of the key regulators for GLUT4 translocation.^(9)^ Hence, we investigated whether CLPr-induced of GLUT4 translocation was differ from the administration timings. CLPr-administration in the rest-phase, but not in the active-phase, increased GLUT4 translocation into the plasma membrane of skeletal muscle in dose-dependent manner without affecting the GLUT4 expression level (Fig. 4). Thus, CLPr-induced GLUT4 translocation, which is a downstream event for AMPK, also has a suitable administration timing.

### Effect of CLPr-administration at different timings on prevention of postprandial hyperglycemia estimated by OGTT

**Fig. 5:**
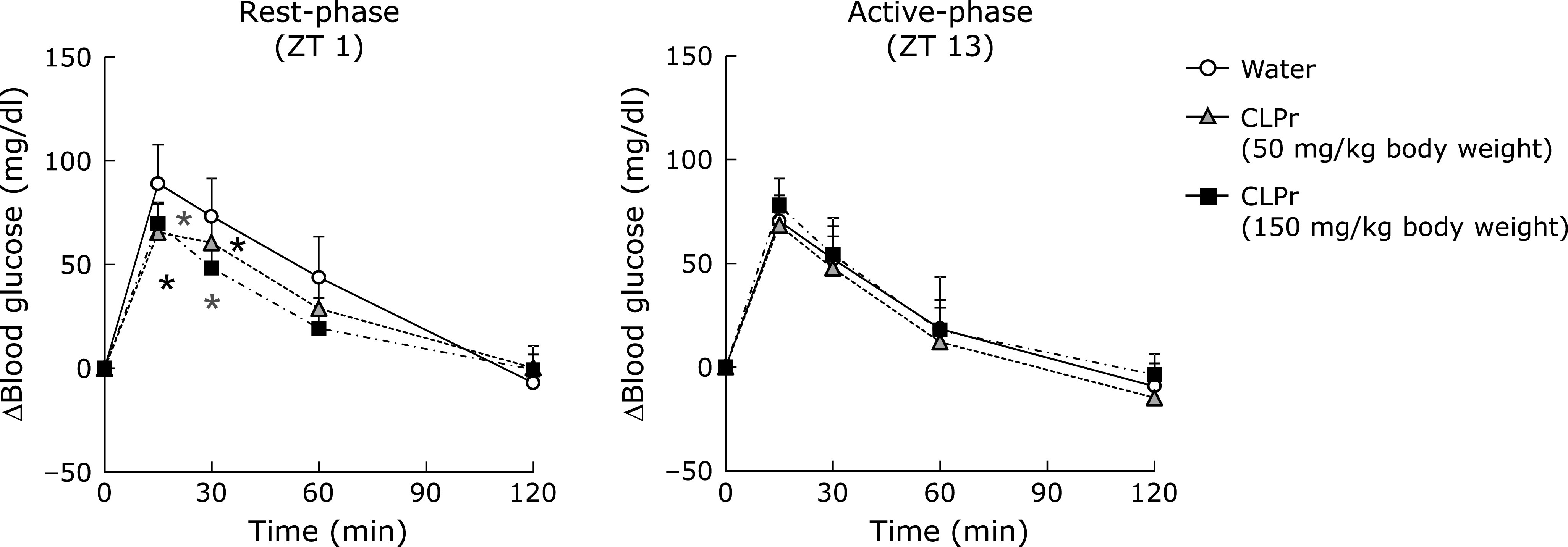
Effects of administration timing of CLPr on the postprandial hyperglycemia. ICR mice were orally administered CLPr at 50 and 150 mg/kg body weight or water (5.0 ml/kg body weight) at ZT1 or 13. Then, OGTT was carried out 1 h after the CLPr administration. The plasma glucose level was measured at 0, 15, 30, 60 and 120 min after the glucose loading. The results are presented as the mean ± SE (*n* = 5). Asterisks indicate significant differences (*p*<0.05, vs water group by Dunnett’s multiple comparison test).

Finally, we investigated prevention effect of CLPr-administration on postprandial hyperglycemia in the rest- and the active-phase. In both phase, glucose-loading expectedly increased the blood glucose level and reached a maximum at 15 min, and then gradually decreased in a time-dependent manner. Pre-administration of CLPr significantly decreased the blood glucose levels at 15 and 30 min after the glucose loading, when OGTT was performed in the rest-phase (Fig. 5A). In contrast, in the active-phase, there were no difference in the blood glucose levels throughout the experimental periods (Fig. 5B). From these results, prevention effect of CLPr on postprandial hyperglycemia also has the suitable administration timing.

## Discussion

Many researches have been reported that certain polyphenols and polyphenol-rich food materials have a potency to prevent hyperglycemia.^(29–32)^ However, the effective and/or suitable administration timing is still unclear yet. Our previous studies have demonstrated that flavan-3-ols-rich CLPr prevents hyperglycemia through AMPK phosphorylation.^(17,19)^ In the present study, we investigated to understand the effective administration timing of CLPr and found that the CLPr-administration at the beginning of rest-phase revealed the effectiveness for prevention of hyperglycemia. Our current results confirmed CLPr increased phosphorylation of AMPK (Fig. 1) and its upstream LKB1 (Fig. 2), resulting in prevention of hyperglycemia (Fig. 4) accompanied by translocation of GLUT4 (Fig. 3). All these events were consistently observed after CLPr-administration at the beginning of rest-phase (ZT1), but not at the beginning of active-phase (ZT13). Our findings support results of the recent report: Takahashi *et al.*^(4)^ demonstrated that the intake of EGCG at the end of active-phase decreased the blood glucose level more effectively compared to the intake of it at the beginning of the active-phase in both mice and human. However, they did not address to the mechanism by which EGCG decreased blood glucose level. In the present study, we confirmed the prevention mechanism of CLPr is coincide with our previous reports.^(17,19)^ Taken together, current and previous findings suggest that flavan-3-ols-caused prevention of hyperglycemia has an effective timing at around ZT0.

AMPK is an important regulator of energy metabolism.^(10,13)^ It is reported that AMPK also regulates circadian rhythm by modulating the expression of several clock gene products in mammalian tissues; e.g., AMPK promotes phosphorylation of CRY1 and CRY2 and stimulates their degradation in the liver.^(33)^ In contrast, AMPK phosphorylation also have circadian rhythm. Basse *et al.*^(34)^ reported that AMPK phosphorylation oscillated and a significant increase in the phosphorylation was observed at ZT13 in the skeletal muscle of mice with free access to an exercise wheel.

In our present study, CLPr administration significantly increased the phosphorylation level of AMPK in skeletal muscle at ZT1, but not at ZT13 (Fig. 2). Recently, we have demonstrated that a single oral administration of CLPr at ZT3 also increased phosphorylation of AMPK and LKB1 in the liver accompanied by increased plasma insulin and glucagon like peptide-1 (GLP-1).^(18)^ In the same previous report, we found GLP-1 antagonist Exendin (9-39) canceled AMPK phosphorylation. Furthermore, it is reported that secretion of GLP-1 has the rhythm and its secretion level in the active-phase is higher than that in the rest-phase.^(35)^ These previous results support the results in the present study. The rhythm of AMPK phosphorylation may be depending upon the stimuli. Relationship between AMPK phosphorylation and circadian rhythm is a controversial issue. Therefore, further study is needed to clarify this controversial issue.

In the present study, we found that CLPr-administration effectively suppressed postprandial hyperglycemia at ZT1, but not at ZT13 (Fig. 5). Phosphorylation of AMPK observed in this study may be contribute to this anti-hyperglycemic effect through promoting GLUT4 translocation (Fig. 1 and 4). Recent studies demonstrate that the circadian system is important to regulating the daily rhythm in glucose metabolism.^(34,36–39)^ Postprandial glucose level, namely glucose spike is influenced by meal timing, in fact, elevated glucose level is higher in the evening than in the morning in human.^(5–7)^ This difference may be related to the secretion level of insulin and its sensitivity. Particularly, insulin sensitivity in mice follows a diurnal variation with the lowest response to insulin during the early light phase.^(40–42)^ Consistently with this result, it is reported that insulin signaling and sensitivity in isolated skeletal muscle peaked during the dark phase in the mice.^(41)^ Taken together, these previous results indicated that glucose tolerance and insulin sensitivity decrease in the rest-phase and increase in the active-one. Our findings in the present study indicate that CLPr improves hyperglycemia during the lowered insulin sensitivity. Moreover, this beneficial effect of CLPr is due to the raised insulin and GLP-1 secretion.^(17)^ Therefore, the intake of CLPr may alleviate the decreased insulin sensitivity through GLP-1 secretion in the evening.

In conclusion, CLPr-suppressed hyperglycemia has a suitable administration timing accompanied by AMPK-induced GLUT4 translocation in mice. For human, the intake of flvan-3-ol-rich food material in the evening is effective to get its health beneficial functions including anti-hyperglycemia.
